# Comparative Transcriptome Profiling Reveals Key MicroRNAs and Regulatory Mechanisms for Aluminum Tolerance in Olive

**DOI:** 10.3390/plants12050978

**Published:** 2023-02-21

**Authors:** Yi Wu, Fangbin Cao, Lupeng Xie, Feibo Wu, Shenlong Zhu, Chengwei Qiu

**Affiliations:** 1 Department of Agronomy, College of Agriculture and Biotechnology, Zijingang Campus, Zhejiang University, Hangzhou 310058, China; 2 Institute of Crops and Nuclear Technology Utilization, Zhejiang Academy of Agricultural Sciences, Hangzhou 310021, China

**Keywords:** Aluminum stress, miRNA, target gene, transcription factor, *Olea europaea* L.

## Abstract

Aluminum toxicity (Al) is one of the major constraints to crop production in acidic soils. MicroRNAs (miRNAs) have emerged as key regulatory molecules at post-transcriptional levels, playing crucial roles in modulating various stress responses in plants. However, miRNAs and their target genes conferring Al tolerance are poorly studied in olive (*Olea europaea* L.). Here, genome-wide expression changes in miRNAs of the roots from two contrasting olive genotypes Zhonglan (ZL, Al-tolerant) and Frantoio selezione (FS, Al-sensitive) were investigated by high-throughput sequencing approaches. A total of 352 miRNAs were discovered in our dataset, consisting of 196 conserved miRNAs and 156 novel miRNAs. Comparative analyses showed 11 miRNAs have significantly different expression patterns in response to Al stress between ZL and FS. In silico prediction identified 10 putative target gene of these miRNAs, including MYB transcription factors, homeobox-leucine zipper (HD-Zip) proteins, auxin response factors (ARF), ATP-binding cassette (ABC) transporters and potassium efflux antiporter. Further functional classification and enrichment analysis revealed these Al-tolerance associated miRNA-mRNA pairs are mainly involved in transcriptional regulation, hormone signaling, transportation and metabolism. These findings provide new information and perspectives into the regulatory roles of miRNAs and their target for enhancing Al tolerance in olives.

## 1. Introduction

Aluminum (Al) is the third most abundant element in the Earth’s crust, after oxygen and silicon [1]. Under acidic soils condition (pH < 5), Al in harmless precipitates tends to dissociate as the phytotoxic trivalent Al^3+^, which leads to inhibition of root growth, disruption of water and nutrients uptake, finally limiting the yield of crops [2]. Acidic soil occupies about 40–50% of cultivable area throughout the world [3]. To survive in acidic soils with high content of active Al, plant species have developed a variety of tolerant mechanisms, including Al^3+^ exclusion and internal detoxification strategies [4]. A number of genes such as *ALMT1* (Al-activated malate transporters), *MATE* (multidrug and toxic compound extrusion) and *ALS3* (aluminum sensitive 3) have been recognized as important elements for Al tolerance [5,6,7]. However, our understanding of novel genes conferring Al tolerance and molecular mechanisms underlying these processes is still limited.

Plant non-coding RNAs (ncRNAs) are functional transcripts with low protein-coding potential, including miRNAs, siRNAs, lncRNAs and circRNAs, which play an important role in plant development, hormone signaling, and abiotic stress responses [8,9]. MicroRNAs (miRNAs), a class of endogenous, non-coding, short, single-stranded RNA molecules, are widely distributed across eukaryotes. The highly conserved and tissue-specific miRNAs are master regulators of gene expression in plant, which guide degradation of the mRNA targets, and subsequently suppress expression of the target genes [10]. Recently, global expression pattern of miRNAs in response to Al stress have been conducted in several species. A total of 23 Al-responsive miRNAs were identified in the leguminous model plant *Medicago truncatula*, and most of these miRNAs were dynamically down-regulated during the treatment [11]. The miRNA expression profiles of cultivated soybeans revealed that conserved miRNAs, such as gma-miR166k/o, gma-miR390g, and gma-miR396c/k, were involved in root elongation for Al-tolerance [12]. The miRNA-mediated regulation of root elongation was also discovered in *Arabidopsis* and barley under Al stress [13,14]. The miR166b/*HvHOX9* module was identified and functionally characterized as a crucial regulatory factor for Al detoxification in Tibetan wild barley [15]. Nevertheless, the involvement of miRNAs in Al stress-induced response and their regulatory networks in olive are largely unknown.

Olive (*Olea europaea* L.) is one of the most important oil fruit tree crops worldwide. In recent few years, the requirement of heathy diet resulted in a rapid growth in olive oil consumption. The olive cultivation is traditionally concentrated in the Mediterranean area with dry summers and neutral/alkaline soils. Since 1960s, China has been a newly emerging olive-oil-producing region in the world [16]. However, due to the huge climate and soil differences with the Mediterranean region, the large-scale introduction and cultivation of most olive cultivars in southern China are hampered by the acidic soil and Al toxicity, resulting in lower yield and fruit quality. Thus, more studies are necessary to exploit elite genetic resources and elucidate the mechanism of Al detoxification for improvement of high-tolerance cultivars.

Here, we performed genome-wide investigation of Al stress responsive miRNAs of the two contrasting olive genotypes ZL (Al-tolerant) and FS (Al-sensitive) using high-throughput RNA-sequencing approach. Small RNA libraries from roots of both genotypes exposed to Al stress and without Al treatment were constructed. Global expression pattern profiling and comparative analyses were conducted, followed by functional prediction. The objective of the present study is to identify key miRNAs and their target genes associated with Al tolerance in olive, provide molecular basis for illustration of the underlying regulatory mechanisms, and extend our knowledge on genetic improvement of olive.

## 2. Results

### 2.1. Genome-Wide Identification of Small RNAs in Two Olive Genotypes

**Figure 1:**
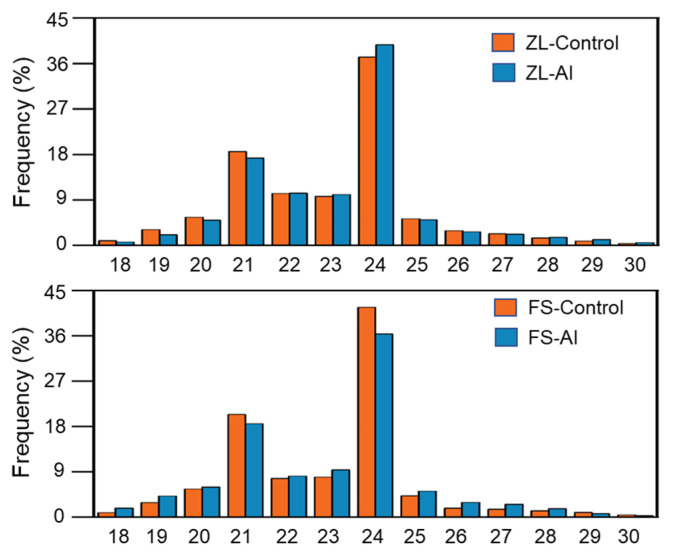
Length distribution of small RNAs in roots of ZL and FS. The X-axis represents the small RNA length (nucleotide) and the Y-axis represents the percentage of small RNA reads. Control corresponds to hydroponically grown in basic nutrition solution and Al represents 50 µM Al treatment for 24 h.

Four small RNA libraries (ZL-Control, ZL-Al, FS-Control and FS-Al) were constructed from roots of two olive genotypes under normal and Al-treatment condition. In total, 29,566,134, 29,626,516, 29,635,826 and 29,637,498 raw reads were generated by BGI-500 platform for these four libraries, respectively (Table S1). After the removal of low quality and contaminated reads, 27,011,675, 27,136,823, 26,674,901 and 26,916,653 clean reads were obtained, accounting for 91.36%, 91.60%, 90.01% and 90.82% of the raw reads, respectively. These clean data were mapped onto the genome of *Olea europaea* L., 18,815,636 (69.66%), 17,398,002 (64.11%), 21,131,762 (79.22%) and 17,398,002 (64.11%) were matched to the reference genome in the four RNA libraries, respectively. The Q20 percentage of these clean tags was more than 99%, indicating a robust quality of deep sequencing and data filtering. Upon screening the size of these sequences, most clean reads were further confirmed as small RNA (sRNA) molecules. The length distribution of sRNAs in four libraries was summarized in Figure 1. The most abundant sRNAs ranged from 19–25 nt, and the 24 nt sRNAs represented the most frequent size. These results were most extensively identical among those reported for other plants, suggesting the reliability of our dataset.

### 2.2. Identification of Conserved miRNAs and Novel miRNAs

**Figure 2:**
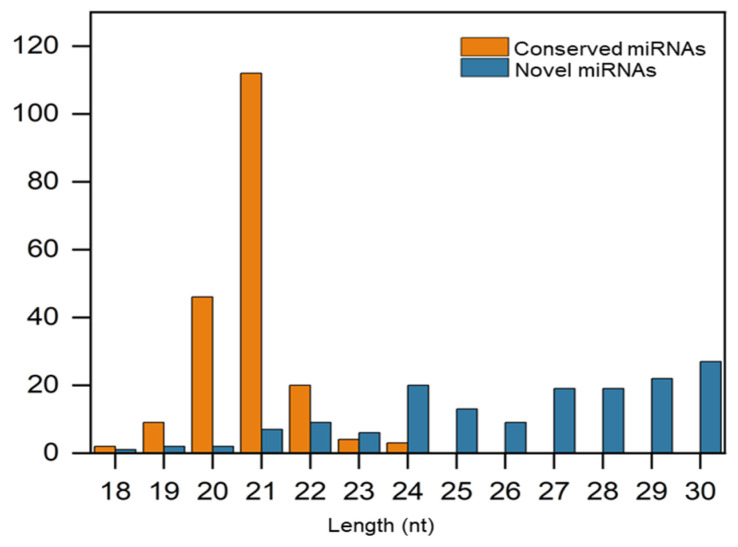
Length based distribution of conserved and novel miRNAs in olive roots.

To investigate the global profiles of miRNAs in olives, all valid clean sRNA reads from four libraries were submitted to miRBase (Ver 22.0) for BLAST analysis. A total of 196 unique conserved miRNAs were identified in our dataset. Among them, the most abundant length was 21 nt, accounting for 57.14% of the total miRNAs, followed by 20 nt (23.47%) and 22 nt (10.20%), and the least abundant length was 18 nt (1.02%) (Figure 2).

**Figure 3:**
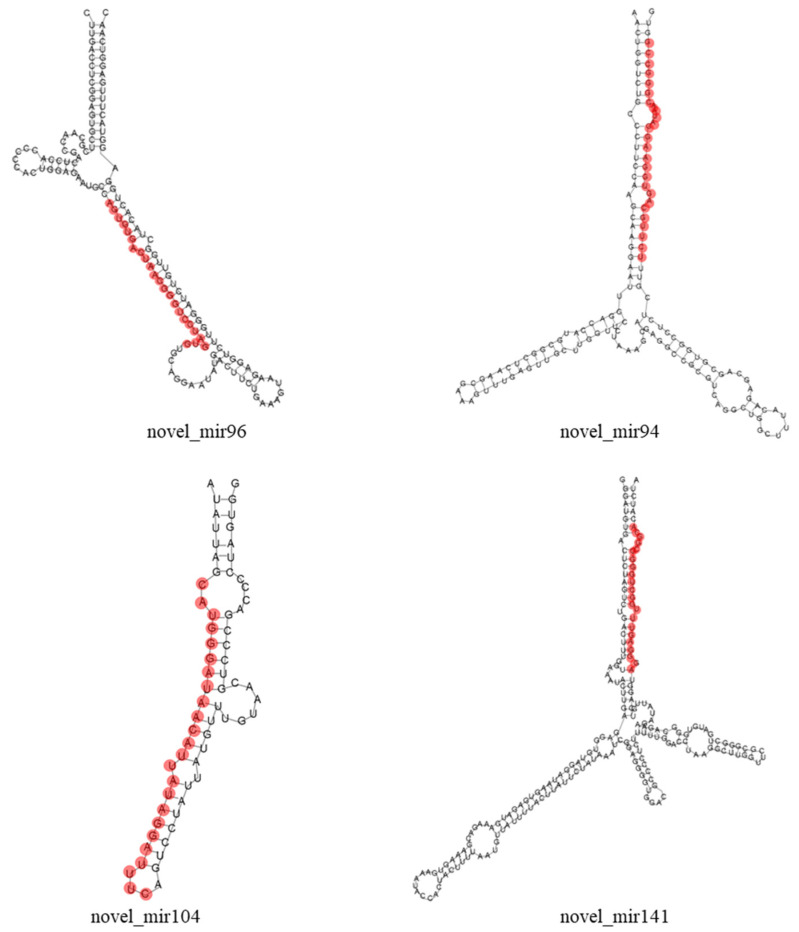
Precursor structure prediction of four novel miRNAs differently expressed in ZL and FS in response to Al stress. Red bars indicate the sequence of mature miRNAs.

Based on the recent annotation criteria, novel miRNAs in olive were predicted by miRA software. A total of 156 sRNA unique sequences were identified as putative novel miRNAs. These predicted novel miRNAs ranged from 18 to 30 nt in length, the most abundant length was 30 nt, accounting for 17.31% of the total novel miRNAs, followed by 29 nt (14.10%) and 24 nt (12.82%), while the least abundant length was 18 nt (0.64%) (Figure 2). In addition, secondary structure prediction of four novel miRNAs differentially expressed in ZL and FS in response to Al stress is shown in Figure 3.

### 2.3. Genotypic Differences in miRNA Expression Profiles in Response to Al Stress

**Figure 4:**
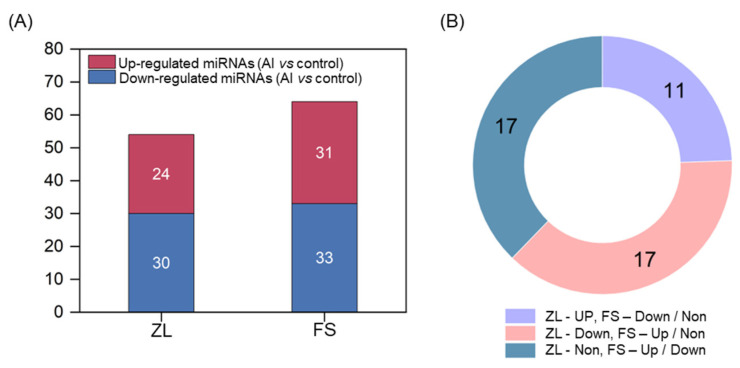
Al stress-responsive miRNAs in two olive genotypes. (**A**) Number of up- and down-regulated miRNAs of ZL and FS in response to Al stress. (**B**) Comparative analysis of Al stress-responsive miRNAs in two olive genotypes. Fold change (Al vs. control) is log_2_N, log_2_N ≥ 1 are up-regulated (Up), between −1 < log_2_N < 1 are unchanged (Non) and log_2_N ≤ −1 are down-regulated (Down), Adjusted *p*-value ≤ 0.01.

**Figure 5:**
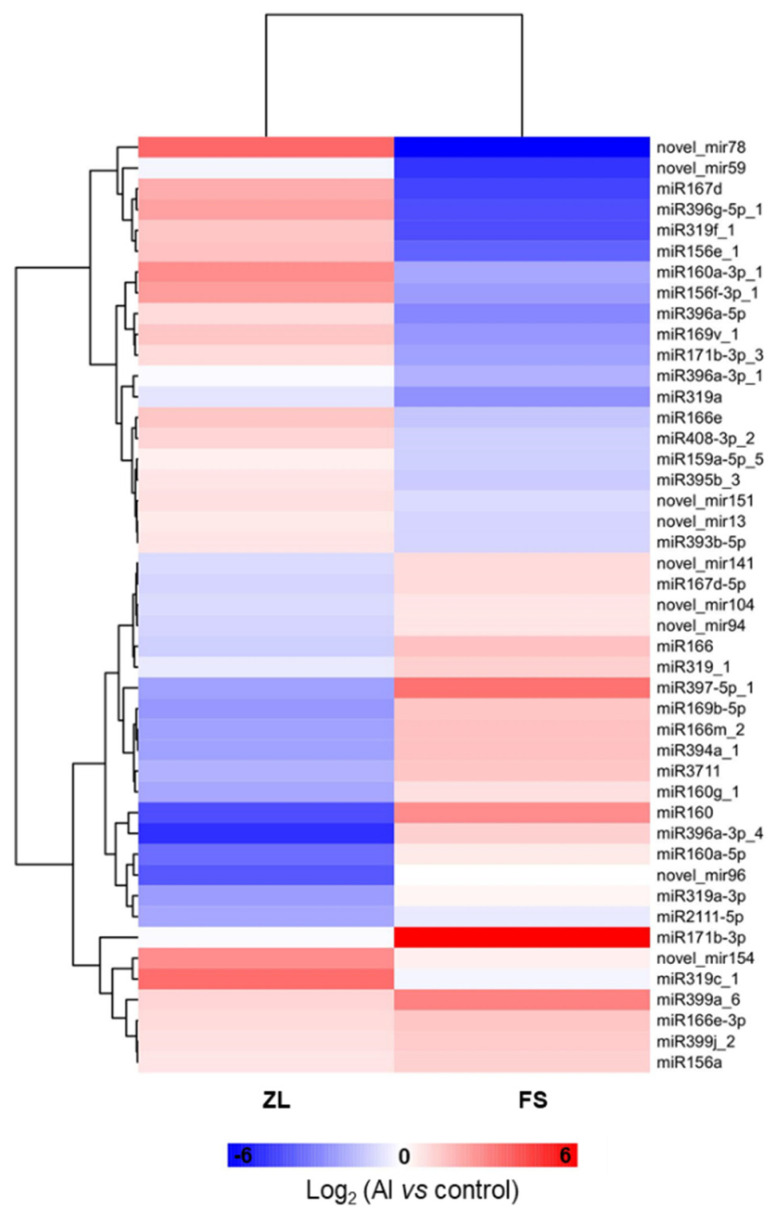
The hierarchical clustering analysis of Al-responsive miRNAs from roots of ZL and FS. Hierarchical clustering of differently expressed miRNAs was displayed by Euclidean distance and complete cluster methods as a measurement of similarity.

To identify Al-stress responsive miRNAs in olive, the expression level of each miRNA was normalized by TPM (transcripts per million) values from the four libraries. Paired comparative analyses were subsequently performed for determine differentially expressed miRNAs by DEGs analysis software. Compared with the control group (Al vs. control), 30, 24 and 138 miRNAs were up-regulated, down-regulated, and unchanged in response to Al stress in ZL, respectively (Table S3); and the corresponding figures for FS were 33, 31 and 142 (Table S4). According to genotypic differences in the expression profile between ZL and FS, 45 miRNAs were characterized as Al-tolerance associated miRNAs (Table S2). Among them, 11 miRNAs were up-regulated in ZL but down-regulated or remained unchanged in FS; 17 miRNAs were down-regulated in ZL but up-regulated or remained unchanged in FS; and 17 miRNAs were unchanged in ZL but up- or down- regulated in FS (Figure 4A,B; Table S5). The hierarchical clustering analysis of these miRNAs from roots of ZL and FS revealed the significantly genotype-specific expression patterns in response to Al stress (Figure 5). According to the negative regulation between miRNAs and their target genes, we focus on miRNAs that were down-regulated in ZL, but non-changed or up-regulated in FS; and non-changed in ZL, but up-regulated in FS. Therefore, 19 miRNAs were recognized as key miRNAs for Al tolerance and were further investigated (Table 1).

### 2.4. Functional Characterization of Key miRNAs and Their Target Genes

To comprehend how miRNAs participate in regulation of Al tolerance in olive, psRobot and TargetFinder were employed to predict target genes for differentially expressed miRNAs in both ZL and FS, followed by GO (Gene Ontology) classification and KEGG (Kyoto Encyclopedia of Genes and Genomes) enrichment analyses.

**Figure 6:**
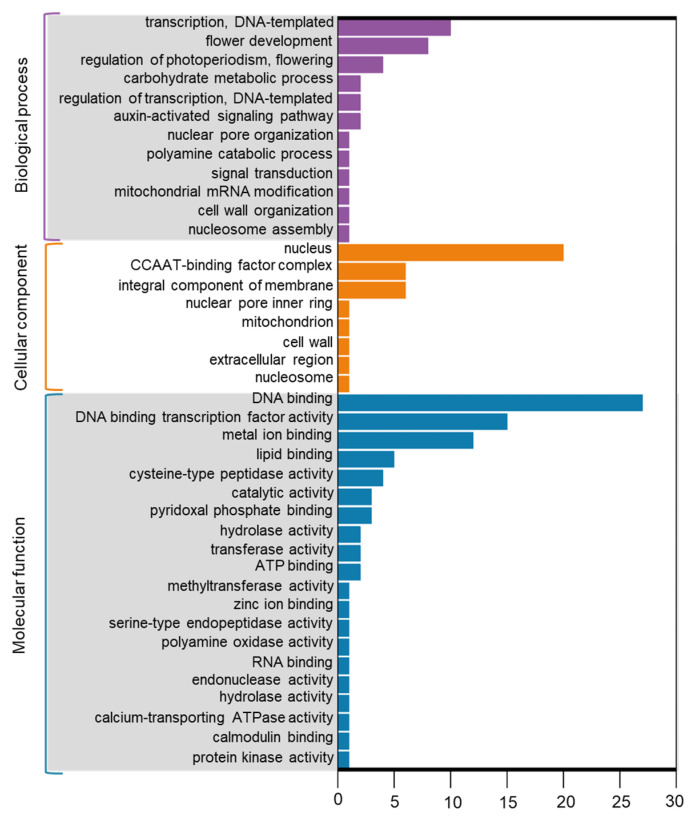
Gene Ontology (GO) analysis for target genes of differently expressed miRNA in ZL and FS in response to Al stress.

**Figure 7:**
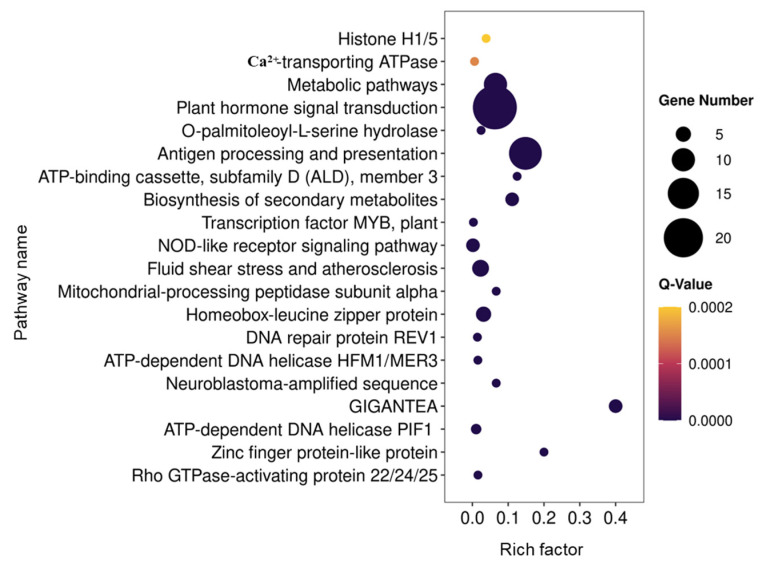
Kyoto Encyclopedia of Genes and Genomes (KEGG) enrichment analysis for target genes of differently expressed miRNAs in ZL and FS in response to Al stress.

The prediction of differentially expressed miRNA target genes revealed that most target genes encode transcription factors, such as the homeobox-leucine zipper protein ATHB-15, MYB33, GAMYB, and nuclear transcription factor Y subunit A. In addition, annotation of other key target genes consisted of ABC transporter family, K^+^ efflux antiporter, rho GTPase-activating protein and dehydration-responsive element-binding protein (Table S5). GO analysis showed the target genes of differentially expressed miRNAs are mainly involved in DNA binding, DNA-binding transcription factor activity and metal ion binding in molecular functions; nucleus, CCAAT- binding factor complex and integral component of membrane in cellular components; and transcription, DNA-templated, flower development, regulation of photoperiodism and flowering in biological processes (Figure 6). According to the KEGG enrichment analysis, the target genes of differentially expressed miRNAs in both ZL and FS were mainly concentrated in pathways, such as plant hormone signal transduction, antigen processing and presentation, metabolic pathways, fluid shear stress and atherosclerosis and homeobox-leucine zipper protein pathways (Figure 7). These results suggested that miRNAs and their target genes in different biological functions and pathways might play crucial roles in Al stress tolerance.

## 3. Discussion

**Figure 8:**
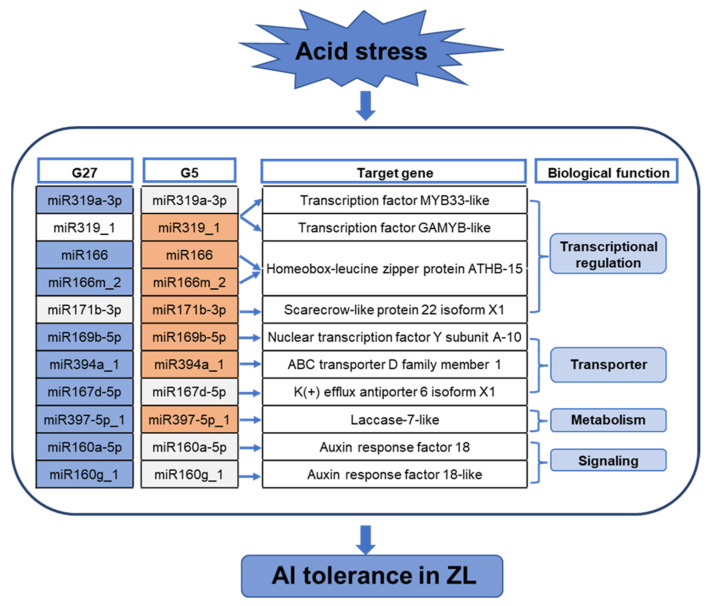
Hypothetical regulatory model involving miRNA and their target genes in Al-tolerance of ZL. Colors in the heatmap represent the up-regulated (orange), unchanged (grey) or down-regulated (blue) of each miRNA in response to Al stress.

Recent high-throughput sequencing researches have revealed a number of stress responsive miRNAs in several plant species under multiple adverse conditions, and have greatly advanced our knowledge of the miRNA functions in stress tolerance [17,18]. In the present study, a comprehensive study was conducted using two olive genotypes, ZL and FS, that differ in Al stress responses to identify the key miRNAs and target genes conferring Al tolerance and investigate their involvement in regulatory mechanism against Al toxicity. We identified 11 miRNA–mRNA pairs were significantly differently expressed between ZL and FS, which confer Al resistance by regulation of gene expression, hormone signaling, transportation and metabolism in ZL (Figure 8).

### 3.1. miRNA–mRNA Pairs Mediate Al Tolerance by Transcriptional Regulation and Hormone Signaling

MYB proteins comprise one of the largest transcription factor families in plants and play an important role in various abiotic stresses in plant [19]. For instance, the MYB transcription factor mediated by miR159 has been shown to be an essential element in wild soybean in response to Al stress [20]. MYB103 was also considered as the regulator of Al sensitivity in *Arabidopsis thaliana* by modulating the *O*-acetylation level of cell wall xyloglucan [21]. In alfalfa, MsMYB741 positively activated the expression of downstream genes, and increased flavonoid accumulation in roots and secretion from root tips, leading to enhanced Al resistance [22]. In this study, MYB33 and GAMYB were found to be the target of miR319a-3p and miR319_1. The up-regulation of these MYB transcription factors mediated by miRNAs may increase the tolerance to Al stress in olive.

HD-Zip protein family is also key regulators that responsible for plant growth and development as well as environmental adaptation. In *Medicago truncatula*, MIR166-mediated *HD-Zip III* genes were expressed predominantly in root vascular system as well as in the lateral root primordia, which was essential for legume root architecture [23]. In *Arabidopsis*, two HD-Zip I transcription factors were also identified and functionally characterized to be involved in regulation of root growth and Al tolerance [24]. Moreover, Feng et al. reported a novel HD-Zip transcription factor gene *HvHOX9*, which positively regulates Al tolerance in Tibetan wild barley through increases rhizosphere pH and decreasing root cell wall Al binding capacity [15]. In this study, we also found the target gene of miR166 and miR166m_2 encodes a member of HD-Zip protein, suggesting a potential role in modification of olive root system under Al stress.

GRAS family members constitute a group of plant-specific transcription factors that are named after the GIBBERELLIN-INSENSITIVE (GAI), REPRESSOR of GAI (RGA) and SCARECROW (SCR). Among them, scarecrow-like (SCL) protein are essential for root cell radial patterning, the quiescent center maintenance and the endodermis differentiation in many plants [25]. Mendoza-Soto et al. [26] found miR170–SCL pair in soybean regulates plant tolerance to Al toxicity by participating in the gibberellin signaling pathway. Here, SCL protein 22 isoform X1 was predicted as the target gene of miR171b-3p, which may also positively regulate Al stress tolerance in olive.

ARFs bind with specificity to auxin response element (AuxRE) TGTCTC sequence in the promoter of primary/early auxin-response gene, which directly related to many physiological processes, including formation of flower organs and vascular bundle tissues, differentiation of xylem and phloem, root generation, leaf senescence, fruit maturation and development, and regulation of tropism and apical dominance [27]. Okushima et al. [28] found that *ARF7* and *ARF19* genes enhanced auxin signaling during lateral root development of *Arabidopsis*. *ARF6* and *ARF8* were also involved in formation of adventitious roots with the participation of miR160 and miR167 [29]. In this study, the target genes of miR160a-5p and miR160g_1 were annotated as auxin response factor 18. The up-regulated ARFs might improve the aluminum tolerance of olive by participating in lateral root formation through auxin signaling pathways.

### 3.2. miRNA–mRNA Pairs Mediate Al Tolerance by Regulation of Transportation and Metabolism

Nuclear factor-Y (NF-Y) transcription factors are ubiquitous among eukaryotes. Some members of the NF-Y family are induced by stress and regulated by miR169 [30]. Many studies have demonstrated the important role of NF-Y family in abiotic stress, particularly in drought, salt and high temperature stress [31,32,33]. NF-YA is closely related to stress response and signal transduction genes [34,35]. We found the target gene of miR169b-5p is associated with NF-Y. Therefore, it may be involved in the regulation of Al transporters, and enhance the absorption and resistance of olive to Al.

ABC transporter superfamily are ubiquitous among living organisms. In plant, ABC family members were initially found to be transporters responsible for the detoxification mechanisms. For example, both ABC transporter genes *STAR1* (sensitive to Al rhizotoxicity 1) and *STAR2* were identified as Al-tolerance genes in rice, and the mutation in *star1* and *star2* resulted in hypersensitivity to Al stress [36]. Another gene *OsALS1*, encoding a tonoplast-localized ABC transporter, is induced by Al treatment and responsible for the sequestration of aluminum in vacuoles, which is necessary for Al detoxification in rice [37]. ABC transporter B family member 25 were also identified and functionally characterized in barley under Al stress [3]. In this study, the target gene of miR394a_1 was annotated as ABC transporter D family member 1. We found that miR394a_1 was down-regulated in ZL, but up-regulated in FS, indicating a similar role of the ABCD1 gene in enhancing Al tolerance in olive.

Potassium (K^+^) transporters play an important role in plant nutrition, growth, development and signal transduction by regulating intracellular K^+^ homeostasis. Among them, K^+^ efflux antiporters (KEAs) provide a means for acidification of the cytosol as a defense to toxic electrophiles [38]. In *Arabidopsis*, chloroplast-localized K^+^/H^+^ antiporters AtKEA1, AtKEA2 and AtKEA3 play an important role in osmotic adjustment, photosynthesis and pH regulation [39,40]. Here, the target gene of miR167d-5p was predicted as K^+^ efflux antiporter 6 isoform X1, which may be involved in ion homeostasis and osmotic adjustment under Al stress in olive.

Laccases (LAC) belong to the blue copper oxidase/p-diphenol:dioxygen oxidoreductase family and have been documented to be involved in processes of cell elongation, lignification and stress response in plants [41]. In rice, overexpression of miRNA OsmiR397 repressed its target *OsLAC*, leading to increased grain size, panicle branching, and improved rice yield [42]. In *Arabidopsis*, miR397 also positively regulated seed yield by mediating LAC genes [43]. However, we found miR397-5p_1 was down-regulated in ZL, but up-regulated in FS. More investigations are required to elucidate the roles of its target laccase-7-like in Al stress response of olive.

## 4. Materials and Methods

### 4.1. Plant Materials and Treatments

Two olive genotypes Zhonglan (ZL, Al tolerant) and Frantoio selezione (FS, Al sensitive) identified by Niu et al. [44] and grown in the experimental field of Zhejiang Academy of Agricultural Sciences in a 2.0 × 3.0 m space on average and propagated by cuttage in perlite as described by Niu et al. [44]. A greenhouse hydroponic experiment was conducted on Zijingang Campus, Zhejiang University, Hangzhou, China using the cuttings of ZL and FS with new roots of 1.0–3.0 cm and two leaves (about 3 months after propagation). The rooted cuttings were cultured in 1/2 modified Hoagland solution under long-day conditions (16 h light/8 h dark cycle) at 26 °C. Then, uniform rooted cuttings of each genotype were separated averagely into two groups, including Al-treatment group in a 0.5 mM CaCl_2_ and 50 μM AlCl_3_ (pH 5) solution and control group with 0.5 mM CaCl_2_ solution (pH 5). The experiment was arranged in a completely randomized design with four replicates. Each genotype contained at least 30 rooted cuttings as a biological replicate.

### 4.2. RNA Isolation, Library Construction and High-Throughput Sequencing

After 24 h Al exposure, the roots of cuttings were sampled, immediately frozen in liquid nitrogen, and cryopreserved at −80 °C. All reagents were analytical grade and all stock solutions were prepared with DEPC-treated water. Total RNA was extracted from olive roots using TRIzol reagent (Invitrogen, Carlsbad, CA, USA). The quantity and quality of extracted RNA were evaluated using an Agilent 2100 bioanalyzer (Agilent Technologies, Santa Clara, CA, USA). Small RNAs with 18–30 nt in length was separated from total RNA by polyacrylamide gel electrophoresis (PAGE). Then ends of the selected molecules was ligated with adaptors, followed by the generation of first strand of cDNA. The cDNA was amplified using high-fidelity polymerase, PCR products with 100–120 bp were subsequently separated by PAGE electrophoresis. After quantification and pooling, the cDNA libraries were sequenced by BGISEQ-500 platform according to the manufacturer’s instructions [45].

### 4.3. Identification and Expression Analysis of sRNAs in Olive

After removing the adaptor contaminants and low-quality reads, credible clean reads of sRNA sequences were obtained. The clean reads were further matched with non-coding RNA sequences in the GenBank and Rfam database to eliminate tRNA, rRNA, snoRNA and snRNA using AASRA [46] and cmsearch [47]. The remaining reads were traversed and annotated in the following order: miRbase > pirnabank > snoRNA> Rfam > other sRNAs, to ensure that each unique sRNA has a unique annotation. The conserved miRNAs were identified through BLASTn approaches against miRBase database with up to two mismatches; novel miRNAs were predicted by miRA [48]. The expression level of miRNAs was normalized by Transcript Per Million (TPM) methods [49]. The differentially expressed miRNAs were determined by DEGseq software with a threshold of Fold Change ≥ 1 and Adjusted *p*-value ≤ 0.01 [50].

### 4.4. Target Gene Prediction and Functional Analysis

The psRobot and TargetFinder software were used to predict putative target genes for conserved and novel miRNAs using default parameters [51]. All predicted target genes of differently expressed miRNAs were studied by using GO::TermFinder for GO functional annotation [52] and the KEGG public database for pathway enrichment analysis [53]. 

## Figures and Tables

**Table 1:** MicroRNAs associated with Al-tolerance in two contrasting olive varieties.

miRNA ID	Sequence	Fold Change		Target Gene	Annotation
		ZL	FS		
miR160	UGGCAUACAGGGAGCCAGGCA	−4.6	2.7	XM_022996383.1	Probable methyltransferase PMT5 isoform X3 [*S. indicum*]
miR166	UCGGACCAGGCUUCAUUCCCCC	−1.4	1.4	XM_022988900.1	Homeobox-leucine zipper protein ATHB-15 [*S. indicum*]
miR3711	UGGCGCUAGAAGGAGGGCCU	−2.1	1.3	XM_023029508.1	Zinc finger protein BRUTUS-like At1g18910 isoform X1 [*S. indicum*]
miR166m_2	CGGACCAGGCUUCAUUCCCC	−2.5	1.4	XM_022988900.1	Homeobox-leucine zipper protein ATHB-15 [*S. indicum*]
miR397-5p_1	AUUGAGUGCAGCGUUGAUGA	−2.5	3.4	XM_023032806.1	Laccase-7-like [*N.a tomentosiformis*]
miR394a_1	UUGGCAUUCUGUCCACCUCC	−2.5	1.4	XM_023028746.1	ABC transporter D family member 1 [*S. indicum*]
miR169b-5p	CAGCCAAGGAUGACUUGCCGG	−2.8	1.3	XM_022986682.1	Nuclear transcription factor Y subunit A-10 [*S. indicum*]
miR396a-3p_4	GUUCAAUAAAGCUGUGGGAA	−5.3	1	XM_023010161.1	Rho GTPase-activating protein 3 [*S. indicum*]
miR167d-5p	UGAAGCUGCCAGCAUGAUCUG	−1.2	0.8	XM_023009656.1	K^+^ efflux antiporter 6 isoform X1 [*S. indicum*]
miR160a-5p	UGCCUGGCUCCCUGUAUGCCA	−3.7	0.4	XM_022990902.1	Auxin response factor 18 [*S. indicum*]
miR160g_1	UGCCUGGCUCCUUGUAUGCCA	−2.4	0.7	XM_022986017.1	Auxin response factor 18-like [*S. pennellii*]
miR319a-3p	UUGGACUGAAGGGAGCUCCC	−2.7	0.2	XM_023020858.1	Transcription factor MYB33-like [*P. avium*]
				XM_023031930.1	Transcription factor GAMYB-like [*S. indicum*]
miR2111-5p	UAAUCUGCAUCCUGAGGUCUA	−2.4	−0.7	XM_022985946.1	Unnamed protein product [*C. canephora*]
novel_mir141	AGGGAGUUUGGCUGGGGCGGCA	−1.1	0.9	XM_023012233.1	Uncharacterized protein LOC105165878 [*S. indicum*]
miR156a	UGACAGAAGAGAGUGAGCACA	0.5	1.1	XM_022986496.1	Squamosa promoter-binding-like protein 9 [*S. indicum*]
miR319_1	UUGGACUGAAGGGAGCUCC	−0.8	1.1	XM_023020858.1	Transcription factor MYB33-like [*P. avium*]
				XM_023031930.1	Transcription factor GAMYB-like [*S. indicum*]
miR171b-3p	UUGAGCCGUGCCAAUAUCAC	−0.3	6.3	XM_022990202.1	Scarecrow-like protein 22 isoform X1 [*S. indicum*]
				XM_023012724.1	Probable E3 ubiquitin ligase SUD1 [*S. indicum*]
miR166e-3p	CUCGGACCAGGCUUCAUUCCC	0.8	1.4	XM_022988900.1	Homeobox-leucine zipper protein ATHB-15 [*S. indicum*]
miR399j_2	UGCCAAAGGAGAGUUGCCCUA	0.6	1.2	XM_022991328.1	Mitochondrial-processing peptidase subunit alpha-like [*S. indicum*]
miR399a_6	UGCCAAAGGAGAAUUGCCCUG	1.0	3.0	XM_023034395.1	Dehydration-responsive element-binding protein 2A-like [*N. attenuata*]

Fold change (Al vs. control) is log_2_N, log_2_N ≥ 1 are up-regulated, between −1 < log_2_N < 1 are unchanged and log_2_N ≤ −1 are down-regulated, *p*-value ≤ 0.01.

## Data Availability

The data presented in this study are available in article and Supplementary Materials.

## Supplementary Materials

The following supporting information can be downloaded at: https://www.mdpi.com/article/10.3390/plants12050978/s1, Table S1: Summary of high-throughput sequencing of small RNAs from olive roots; Table S2: Summary of differentially expressed miRNA from olive roots in ZL and FS; Table S3: Identification of Al stress responsive miRNAs in ZL; Table S4: Identification of Al stress responsive miRNAs in FS; Table S5: Comparison of Al stress responsive miRNAs in two olive genotypes.
