# Supplementary material for: Comparative Transcriptome Profiling Reveals Key MicroRNAs and Regulatory Mechanisms for Aluminum Tolerance in Olive
Source: Plants (Basel). 2023 Feb 21;12(5):978. doi: 10.3390/plants12050978 (PMC10005091; doi:10.3390/plants12050978)
Supplement: Supplementary file 1 [file plants-12-00978-s001.zip › plants-2233501-supplementary.pdf]

Table S1. Summary of high-throughput sequencing of small RNAs from olive roots.

Library Type	ZL		FS	
	Control	Al	Control	Al
Total raw reads	29,566,134	29,626,516	29,635,826	29,637,498
Total clean reads	27,011,675	27,136,823	26,674,901	26,916,653
Total clean reads ratio (%)	91.36%	91.60%	90.01%	90.82%
Mapped to Genome	18,815,636	17,398,002	21,131,762	20,574,969
Total mapped reads ratio (%)	69.66%	64.11%	79.22%	76.44%
Conserved miRNAs	175	169	172	170
Novel miRNAs	142	144	143	145

Total clean reads ratio (%) = Total clean reads / Total raw reads. Total mapped reads ratio (%) = Mapped to Genome / Total clean reads. ZL-Control and FS-Control correspond to ZL and FS grown in BNS; ZL-Al and FS-Al correspond to ZL and FS grown in BNS with 50 μM $AlCl_3$.

Table S2. Summary of differentially expressed miRNA from olive roots in ZL and FS.

Group	Al *vs* control		Known	Novel	Total
	ZL	FS			
I	Up	Up	4	5	9
II	Up	Non	1	1	2
III	Up	Down	8	1	9
IV	Down	Up	8	0	8
V	Down	Non	2	0	2
VI	Down	Down	2	0	2
VII	Non	Up	6	0	6
VIII	Non	Down	8	3	11

Table S3. Identification of Al stress responsive miRNAs in ZL.

miRNA ID	log$_2$(Al *vs* Control)	*P*-value
**miRNAs up-regulated in ZL**		
miR167d	1.9662	0.0000
novel_mir32	9.9818	0.0000
novel_mir9	5.0266	0.0000
miR156a-5p	1.3781	0.0000
novel_mir39	2.9304	0.0000
miR166e	1.3365	0.0000
miR319f_1	1.2876	0.0054
novel_mir125	4.8305	0.0002
miR396g-5p_1	2.1831	0.0000
novel_mir137	3.3577	0.0000
miR169v_1	1.3580	0.0000
novel_mir154	2.6794	0.0000
miR160a-3p_1	2.6964	0.0000
miR169e_3	2.8271	0.0000
miR156e_1	1.4259	0.0000
miR395a_5	1.7094	0.0000
novel_mir78	3.6095	0.0000
miR319c_1	3.4903	0.0000
miR3627a_2	1.0164	0.0008
miR156f-3p_1	2.3000	0.0010
miR390	2.0506	0.0000
novel_mir90	1.6635	0.0000
novel_mir40	4.8305	0.0002
novel_mir31	1.7651	0.0000
miR319b-5p_2	1.6606	0.0014
miR172c_2	3.1300	0.0098
miR393h	1.4255	0.0000
miR396g-5p	1.0462	0.0000
miR399e_5	1.9076	0.0008
miR399j_1	2.1942	0.0000

miRNA ID	log_2(Al *vs* Control)	*P*-value
**miRNAs down-regulated in ZL**		
miR160	-4.5951	0.0000
miR156	-4.2532	0.0000
miR396a-3p_4	-5.3488	0.0000
miR3711	-2.0736	0.0000
miR166h-3p	-1.4758	0.0000
novel_mir141	-1.0732	0.0000
miR166m_2	-2.5264	0.0000
miR167d-5p	-1.2490	0.0000
miR166	-1.3953	0.0000
miR397-5p_1	-2.4566	0.0000
miR160a-5p	-3.7383	0.0000
miR394a_1	-2.4549	0.0000
miR160g_1	-2.3729	0.0000
novel_mir104	-1.0725	0.0000
miR169b-5p	-2.7710	0.0000
miR319a-3p	-2.6975	0.0000
novel_mir94	-1.2838	0.0000
miR2111-5p	-2.4060	0.0000
novel_mir96	-4.3294	0.0011
miR167c-3p_3	-2.3935	0.0003
novel_mir45	-5.3294	0.0000
miR164a_2	-3.3294	0.0029
miR169h_2	-1.3294	0.0002
miR168-3p	-1.3725	0.0000

Table S4. Identification of Al stress responsive miRNAs in FS.

miRNA ID	$\log_2$(Al *vs* Control)	*P*-value
**miRNAs up-regulated in FS**		
novel_mir32	3.1389	0.0000
miR160	2.7124	0.0000
novel_mir9	5.6711	0.0000
miR156a-5p	5.4876	0.0000
miR171b-3p	6.2526	0.0000
miR396a-3p_4	1.0412	0.0000
miR3711	1.2709	0.0000
miR166e-3p	1.3706	0.0000
novel_mir39	1.6867	0.0000
miR166m_2	1.4445	0.0000
miR166	1.4197	0.0000
miR399j_2	1.2360	0.0000
novel_mir125	10.4948	0.0000
novel_mir137	10.2609	0.0000
miR397-5p_1	3.3704	0.0000
miR399a_6	3.0485	0.0000
miR169k	1.4217	0.0000
novel_mir67	3.1189	0.0000
miR319_1	1.1019	0.0000
miR394a_1	1.4155	0.0000
miR169e_3	1.2374	0.0000
miR395a_5	1.6700	0.0000
miR169d-5p_1	1.7273	0.0000
miR169b-5p	1.3008	0.0000
miR160a-5p_1	4.6643	0.0000
miR156h	4.8801	0.0000
miR156a	1.1359	0.0000
miR169f.1	1.9081	0.0000
miR3627a_2	2.5430	0.0002
novel_mir46	1.1920	0.0002
miR160h_1	4.1025	0.0058
novel_mir80	1.4326	0.0067
miR398a-3p_1	1.1145	0.0092

miRNA ID	log_2(Al *vs* Control)	*P*-value
**miRNAs down-regulated in FS**		
novel_mir13	-1.1923	0.0000
miR396a-3p_1	-2.1524	0.0000
miR167d	-4.8003	0.0000
miR396a-5p	-3.1857	0.0000
miR156	-6.1833	0.0000
miR166h-3p	-1.7060	0.0000
miR171b-3p_3	-2.5574	0.0000
miR168b_1	-1.2585	0.0000
miR166e	-1.5501	0.0000
miR159a-5p_5	-1.3721	0.0000
miR408-3p_2	-1.4027	0.0000
miR319f_1	-4.5454	0.0000
miR396g-5p_1	-4.5535	0.0000
miR169v_1	-2.7019	0.0000
miR319a	-2.9046	0.0000
novel_mir98	-1.0959	0.0000
miR172b-5p_3	-1.2555	0.0000
miR395b_3	-1.5055	0.0000
miR160a-3p_1	-2.4274	0.0000
miR156e_1	-4.0972	0.0000
miR393b-5p	-1.1683	0.0000
miR156_2	-1.2354	0.0000
novel_mir78	-6.5378	0.0000
novel_mir72	-1.4639	0.0000
novel_mir83	-1.2899	0.0000
novel_mir151	-1.1346	0.0000
miR164e-5p	-1.6835	0.0000
novel_mir59	-5.2285	0.0000
miR172d-5p_4	-1.3163	0.0003
miR156f-3p_1	-2.5794	0.0003
miR167a	-1.8424	0.0034

Table S5. Comparison of Al stress responsive miRNAs in two olive genotypes.

miRNA ID	ZL	FS	Putative target	Annotation
**miRNAs down-regulated in ZL and up-regulated in FS**				
miR160	-4.6	2.7	XM_022996383.1	probable methyltransferase PMT5 isoform X3 [*Sesamum indicum*]
miR166	-2.5	1.4	XM_023033880.1	homeobox-leucine zipper protein ATHB-15 [*Sesamum indicum*]
miR3711	-2.1	1.3	XM_023029508.1	zinc finger protein BRUTUS-like At1g18910 isoform X1 [*Sesamum indicum*]
miR166m_2	-2.5	1.4	XM_022988900.1	homeobox-leucine zipper protein ATHB-15 [*Sesamum indicum*]
miR397-5p_1	-2.5	3.4	XM_023032806.1	laccase-7-like [*Nicotiana tomentosiformis*]
miR394a_1	-2.5	1.4	XM_023028746.1	ABC transporter D family member 1 [*Sesamum indicum*]
miR169b-5p	-2.8	1.3	XM_023034476.1	nuclear transcription factor Y subunit A-1-like [*Sesamum indicum*]
			XM_023040847.1	nuclear transcription factor Y subunit A-1-like [*Sesamum indicum*]
			XM_023040848.1	nuclear transcription factor Y subunit A-1-like [*Sesamum indicum*]
			XM_023042536.1	nuclear transcription factor Y subunit A-1 isoform X1
miR396a-3p_4	-5.3	1	XM_023010161.1	rho GTPase-activating protein 3 [*Sesamum indicum*]
**miRNAs down-regulated in ZL and non-changed in FS**				
miR167d-5p	-1.2	0.8	XM_023009656.1	K^+ efflux antiporter 6 isoform X1 [*Sesamum indicum*]
miR160a-5p	-3.7	0.4	XM_022996649.1	auxin response factor 18 [*Sesamum indicum*]
miR160g_1	-2.4	0.7	XM_022996649.1	auxin response factor 18-like [*Solanum pennellii*]
miR319a-3p	-2.7	0.2	XM_023020858.1	transcription factor MYB33-like [*Prunus avium*]
			XM_023031930.1	transcription factor GAMYB-like [*Sesamum indicum*]
			XM_023016833.1	TCP transcription factor CIN2A [*Petrocosmea sinensis*]
			XM_023030926.1	transcription factor TCP4-like [*Sesamum indicum*]
miR2111-5p	-2.4	-0.7	XM_022985946.1	unnamed protein product [*Coffea canephora*]
novel_mir141	-1.1	0.9	XM_023012233.1	uncharacterized protein LOC105165878 [*Sesamum indicum*]
novel_mir104	-1.1	0.5	NA	NA
novel_mir94	-1.3	0.6	NA	NA
novel_mir96	-4.3	-0.2	NA	NA
**miRNAs non-changed in ZL and up-regulated in FS**				
miR156a	0.5	1.1	XM_022986496.1	squamosa promoter-binding-like protein 9 [*Sesamum indicum*]
miR319_1	-0.8	1.1	XM_023020858.1	transcription factor MYB33-like [*Prunus avium*]
			XM_023031930.1	transcription factor GAMYB-like [*Sesamum indicum*]
			XM_023016833.1	TCP transcription factor CIN2A [*Petrocosmea sinensis*]
			XM_023030926.1	transcription factor TCP4-like [*Sesamum indicum*]
miR171b-3p	-0.3	6.3	XM_022990202.1	scarecrow-like protein 22 isoform X1 [*Sesamum indicum*]
			XM_023012724.1	probable E3 ubiquitin ligase SUD1 [*Sesamum indicum*]
miR166e-3p	0.8	1.4	XM_023033880.1	homeobox-leucine zipper protein ATHB-15 [*Sesamum indicum*]
miR399j_2	0.6	1.2	XM_022991328.1	mitochondrial-processing peptidase subunit alpha-like [*Sesamum indicum*]
miR399a_6	1	3	XM_023034395.1	dehydration-responsive element-binding protein 2A-like [*Nicotiana attenuata*]

miRNA ID	ZL	FS	Putative target	Annotation
**miRNAs non-changed in ZL and down-regulated in FS**				
miR319a	-0.8	-2.9	XM_023002756.1	transcription factor GAMYB-like isoform X1 [*Sesamum indicum*]
miR395b_3	0.5	-1.5	XM_022989273.1	paired amphipathic helix protein Sin3-like 2 isoform X1 [*Sesamum indicum*]
miR396a-5p	0.8	-3.2	XM_022998171.1	PREDICTED: factor of DNA methylation 1-like [*Erythranthe guttata*]
miR393b-5p	0.6	-1.2	XM_023013314.1	protein TRANSPORT INHIBITOR RESPONSE 1-like [*Sesamum indicum*]
miR396a-3p_1	-0.3	-2.2	XM_023010161.1	rho GTPase-activating protein 3 [*Sesamum indicum*]
miR171b-3p_3	0.8	-2.6	XM_022990202.1	scarecrow-like protein 22 isoform X1 [*Sesamum indicum*]
miR159a-5p_5	0.3	-1.4	XM_022994469.1	protein GIGANTEA-like [*Sesamum indicum*]
miR408-3p_2	0.9	-1.4	XM_023037301.1	LRR receptor kinase BAK1 [*Sesamum indicum*]
novel_mir13	0.5	-1.2	XM_022998275.1	homeobox-leucine zipper protein ATHB-20-like [*Sesamum indicum*]
novel_mir151	0.7	-1.1	XM_023016268.1	pectin acetylesterase 8 isoform X1 [*Sesamum indicum*]
novel_mir59	-0.4	-5.2	NA	NA
**miRNAs up-regulated in ZL and down-regulated in FS**				
miR167d	2	-4.8	XM_023003370.1	mediator of RNA polymerase II transcription subunit 12 isoform X1 [*Sesamum indicum*]
miR166e	1.3	-1.6	XM_023011037.1	homeobox-leucine zipper protein ATHB-14 isoform X2 [*Sesamum indicum*]
miR169v_1	1.3	-4.5	XM_022986682.1	nuclear transcription factor Y subunit A-10 [*Sesamum indicum*]
			XM_023034476.1	nuclear transcription factor Y subunit A-1-like [*Sesamum indicum*]
			XM_023040847.1	nuclear transcription factor Y subunit A-1-like [*Sesamum indicum*]
			XM_023040848.1	nuclear transcription factor Y subunit A-1-like [*Sesamum indicum*]
			XM_023042536.1	nuclear transcription factor Y subunit A-1 isoform X1 [*Sesamum indicum*]
miR156e_1	1.4	-2.7	XM_022986496.1	squamosa promoter-binding-like protein 9 [*Sesamum indicum*]
miR319f_1	2.7	-2.4	XM_023012846.1	phosphatidylinositol/phosphatidylcholine transfer protein SFH8 [*Sesamum indicum*]
			XM_023030926.1	transcription factor TCP4-like [*Sesamum indicum*]
miR396g-5p_1	1.4	-4.1	XM_022992333.1	katanin p80 WD40 repeat-containing subunit B1 homolog [*Sesamum indicum*]
miR160a-3p_1	3.6	-6.5	XM_022989759.1	histone-lysine N-methyltransferase ATXR3-like isoform X1 [*Sesamum indicum*]
miR156f-3p_1	2.3	-2.6	XM_022993063.1	unnamed protein product [*Coffea canephora*]
novel_mir78	2.5	-1.3	XM_023030235.1	telomere repeat-binding factor 1 isoform X3 [*Sesamum indicum*]
**miRNAs up-regulated in ZL and non-changed in FS**				
miR319c_1	3.5	-0.4	XM_023016833.1	TCP transcription factor CIN2A [*Petrocosmea sinensis*]
			XM_023030926.1	transcription factor TCP4-like [*Sesamum indicum*]
novel_mir154	2.7	0.3	NA	NA
